# Infective endocarditis associated with atopic dermatitis

**DOI:** 10.1002/ccr3.8321

**Published:** 2023-12-21

**Authors:** Yamato Tamura, Takehisa Abe

**Affiliations:** ^1^ Department of Cardiovascular Surgery Nara Prefectural Seiwa Medical Center Nara Japan

**Keywords:** atopic dermatitis, infective endocarditis, methicillin‐sensitive *Staphylococcus aureus*, minimally invasive cardiac surgery

## Abstract

Infective endocarditis caused by atopic dermatitis is common in young patients and has a high potential for causing embolism. Because of the high risk of mediastinitis postoperatively, minimally invasive cardiac surgery could be effective.

## Graphical abstract

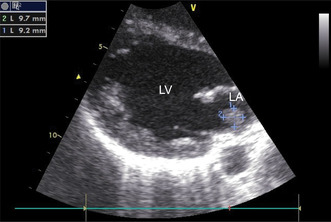

## 1 INTRODUCTION

Without prompt diagnosis and appropriate treatment, infective endocarditis can lead to numerous complications and even death. Causes of bacteremia include infections from intravascular catheters, hemodialysis, and dental procedures; furthermore, recently there have been reports of its connection with atopic dermatitis. Atopic dermatitis is associated with a decreased production of antimicrobial peptides,^1^, ^2^ which are involved in skin defense against infection, rendering the skin susceptible to infection with *Staphylococcus aureus*, a common microbial resident of the skin. Additionally, bacteria can easily invade because of itchiness and scratching.

The incidence of atopic dermatitis among patients with infective endocarditis reportedly ranges from 1% to 7%.^3^, ^4^ It is more common in younger patients, with a mean age of 28.4 years in 8 reported patients; therefore, failure to recognize this association may delay diagnosis.^4^ Among patients with infective endocarditis and atopic dermatitis, the rate of embolism is high, requiring close management and appropriate timing of surgery. There is a high possibility of postoperative mediastinitis after median sternotomy; hence, minimally invasive cardiac surgery (MICS) can be considered.

Herein, we report a patient with infective endocarditis associated with atopic dermatitis who underwent successful MICS with no postoperative wound infection or pyothorax.

## 2 CASE REPORT

An 18‐year‐old man presented to his local doctor with a 5‐day fever and transient loss of consciousness. The loss of consciousness was not preceded by any signs, and no seizure activity was observed before or after the event. Thoracoabdominal computed tomography (CT) and head CT did not indicate any source of fever or cause of loss of consciousness; however, echocardiography showed a suspected verruca at the posterior mitral valve leaflet, prompting referral to our hospital with a diagnosis of infective endocarditis.

The patient had no history of cardiac disease, areas in the oral cavity requiring treatment, or history of drug use that may have contributed to the development of infective endocarditis. However, he had atopic dermatitis since childhood and had an exacerbation of facial erythema 4 months earlier, which subsequently improved 1 month prior.

Upon arrival at our hospital, he was alert but had a temperature of 38.7°C. No paralysis or other neurological abnormalities were noted. His skin condition was not poor, and his abrasions were mild. Osler's nodes were observed in both hands, and the right shoulder was tender, prompting suspicion of a septic shoulder.

**FIGURE 1:**
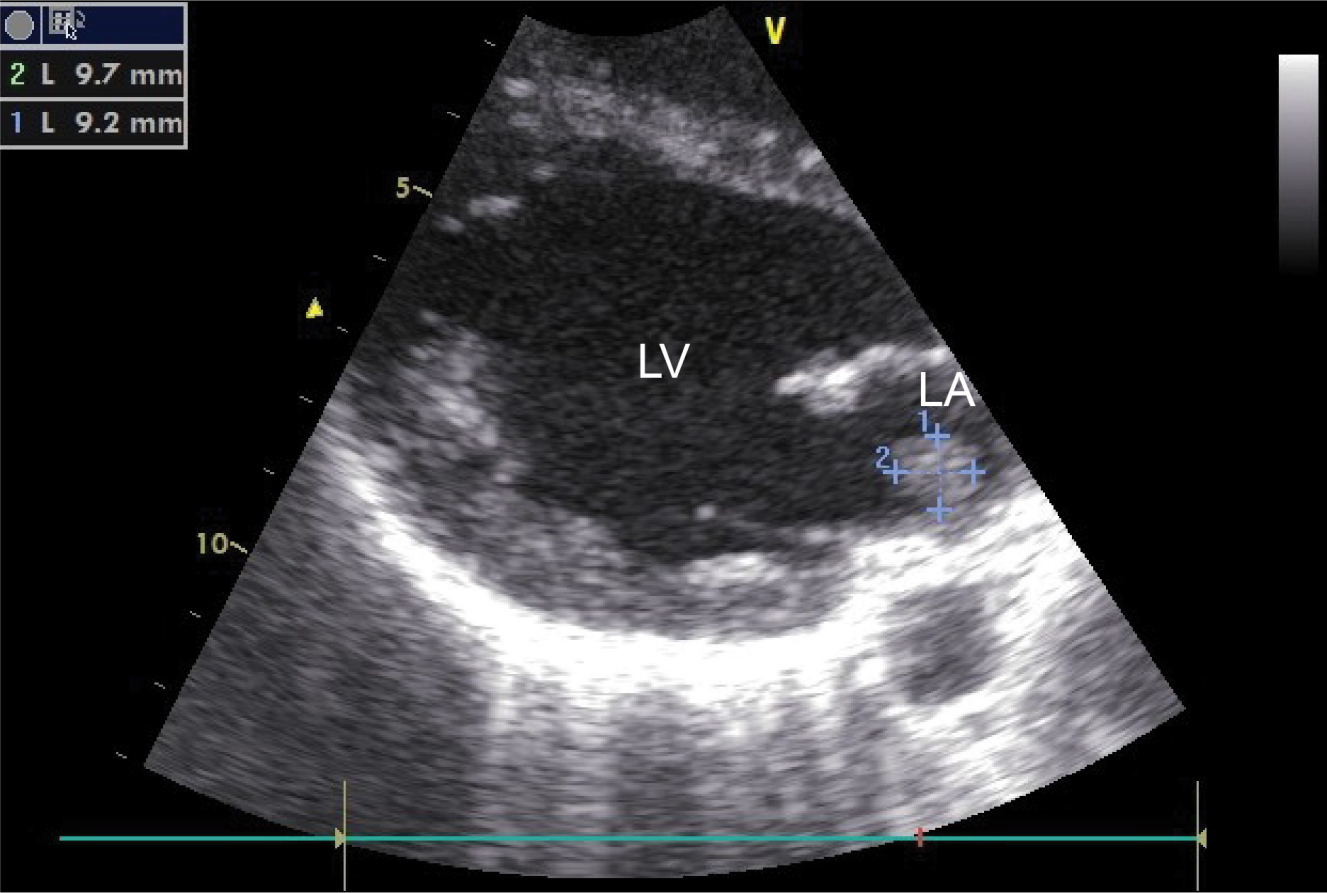
Transthoracic echocardiogram: The verruca was approximately 10 mm in diameter, attached near the posterior mitral valve leaflet and mobile. LA, left atrium, LV, left ventricular.

**FIGURE 2:**
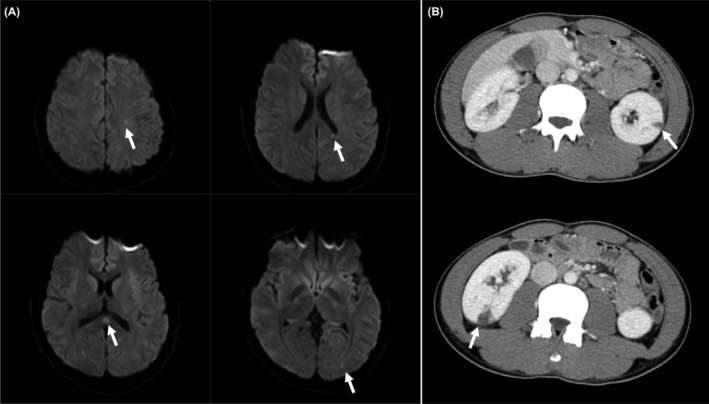
(A) Head magnetic resonance imaging showing multiple cerebral infarcts (arrows). (B) Enhanced computed tomography of the abdomen showing multiple renal infarcts in the bilateral kidneys (arrows).

The blood tests showed a white‐blood cell count (WBC) of 14,700 IU/dL, with neutrophilia but no leftward shift; C‐reactive protein (CRP) level of 14.20 mg/dL; and procalcitonin level of 1.00 ng/mL. There was no anemia, and the liver and renal functions were normal. Blood culture showed methicillin‐sensitive *S. aureus* (MSSA). No other bacteria were detected. Echocardiography showed a verruca on the posterior mitral valve leaflet, which was mobile and about 10 mm in size (Figure 1); mitral regurgitation was mild. Head magnetic resonance imaging (MRI) showed multiple acute cerebral infarctions in the left frontal deep white matter, bilateral occipital lobes, and corpus callosum ampulla (Figure 2). Enhanced CT showed renal infarction and splenomegaly (Figure 2). Spinal MRI showed no evidence of pyogenic spondylitis.

Antibiotic therapy was started, and the patient was referred to our cardiovascular surgery department. Since systemic embolism was observed, we considered further embolism as highly likely and decided on urgent surgery.

**FIGURE 3:**
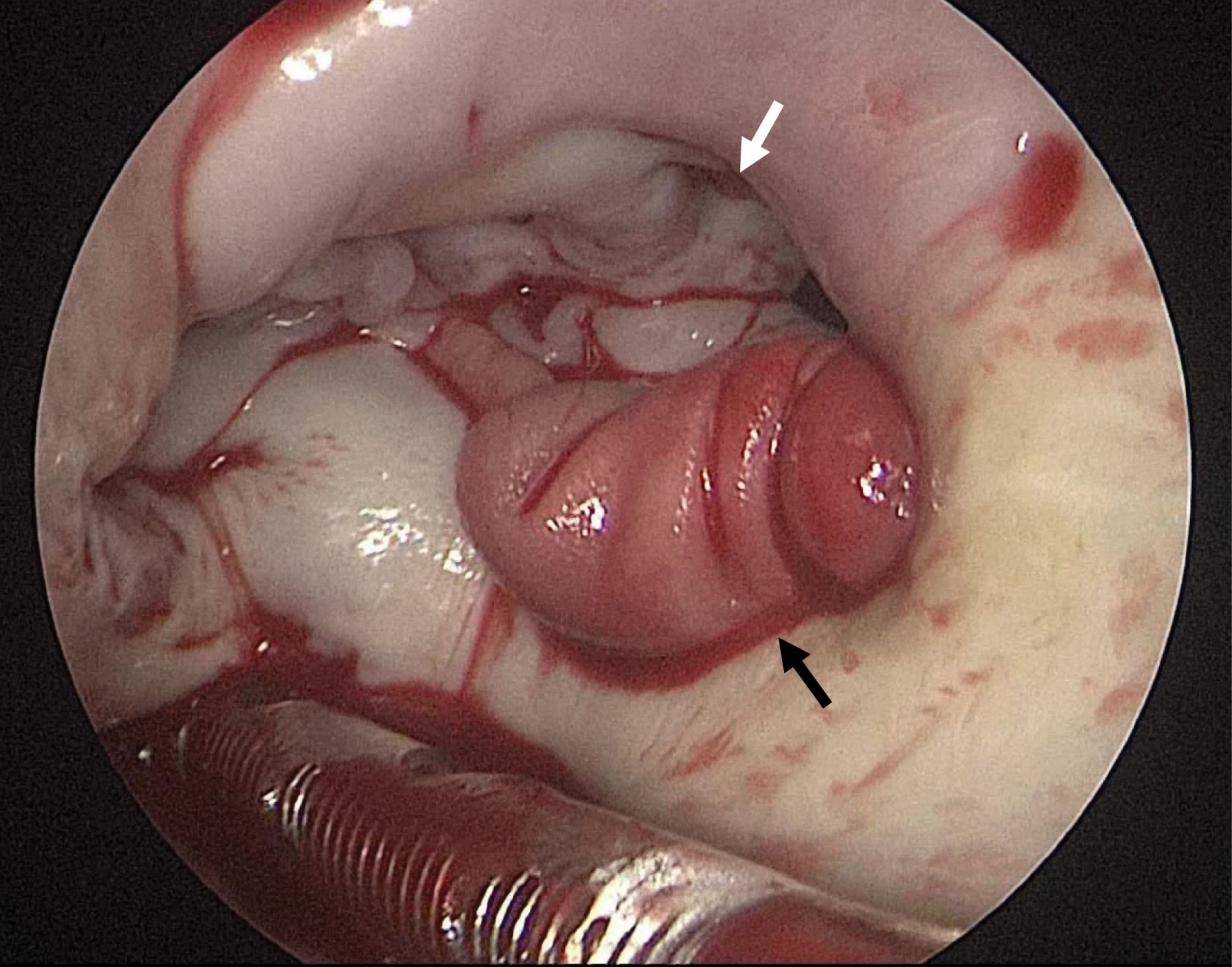
Intraoperative findings: Verrucous lesions on the left atrial wall of the posterior mitral valve annulus (black arrow). There was no destruction of the mitral valve leaflet and annulus. A small verruca was also attached to the anterior mitral valve leaflet (white arrow).

MICS was performed through a small right thoracotomy. A verruca was found on the left atrial wall near the posterior mitral valve annulus. A small verruca was also seen on the anterior mitral valve leaflet (Figure 3). Since there was no destruction of the mitral annulus and leaflet, verrucous excision was performed. Culture of the excised verruca also showed MSSA.

Postoperatively, vancomycin and ceftriaxone were continued for 2 weeks and 6 weeks, respectively. There were no neurological complications, and his general condition improved. His right shoulder pain was relieved, allowing him to elevate his right shoulder. Postoperative head MRI showed no new cerebral infarcts, and the abnormal signals disappeared. Further, there was no evidence of abscess formation or hemorrhage. Enhanced CT of the abdomen showed no significant evidence of renal infarction and no exacerbation. There was no renal dysfunction. Echocardiography also showed no recurrence of verrucae.

The patient was discharged from the hospital after his fever abated. Blood tests showed a WBC count of 7800 IU/dL and a CRP level of 0.05 mg/dL. One year postoperatively, the patient had no recurrence of infection and no complications.

## 3 DISCUSSION

Infective endocarditis is not a disease of high incidence, but when it occurs, it can lead to several complications and even death, requiring prompt diagnosis and appropriate treatment.

In recent years, infective endocarditis associated with atopic dermatitis has been reported. Nakatani et al.^3^ reported that of 513 cases of infective endocarditis, 322 had some background factors, including atopic dermatitis in 5 cases. Fukunaga et al.^4^ reported 120 cases of infective endocarditis, of which 8 were associated with atopic dermatitis (6.7%). In atopic dermatitis, the production of IL‐4, IL‐10, IL‐13, etc. is high, suppressing the production of antibacterial peptides such as β‐defensin and cathelicidin, which are related to skin defense.^1^ Given the synergistic antibacterial activity of β‐defensin and cathelicidin against *S. aureus*, decreased production of these peptides can gradually weaken defense against and enhance susceptibility to *S. aureus* colonization,^5^ rendering the skin susceptible to infection. Consequently, *S. aureus* is detected in more than 90% of patients with atopic dermatitis.^2^ Skin breakage due to scratching may allow bacterial invasion, resulting in bacteremia and infective endocarditis. In particular, patients with atopic dermatitis experience intense itching, which can lead to bacteremia and infective endocarditis caused by *S. aureus* invading through skin abrasions.

Infective endocarditis with a background of atopic dermatitis is common in young patients, and the most common causative organism is MSSA. It is also characterized by a high rate of embolism.^6^ These findings were observed in the present case.

While there have been cases of infective endocarditis related to atopic dermatitis that were successfully managed with antibiotics^7^ and a report of a patient who used dupilumab to improve atopic dermatitis before undergoing standby surgery,^8^ it is crucial to recognize the potential fatality of cerebral infarction resulting from infective endocarditis. Early surgical intervention is necessary when there is a high likelihood of recurrent embolization due to verrucous disease. Some reports suggest that early surgery for infective endocarditis is associated with early death, recurrent infective endocarditis, and valve dysfunction^9^; contrarily, other reports suggest that early surgery is useful for large verrucae.^10^ Infective endocarditis, especially when associated with atopic dermatitis, has been reported to have a high rate of embolism, and early surgery should always be kept in mind. Even right‐sided infective endocarditis, which is considered sensitive to antibiotic therapy, may cause embolization or exacerbation of sepsis during treatment if atopic dermatitis is present in the background.^11^, ^12^ In the present case, the patient had a mobile 10‐mm verruca with a high likelihood of repeated embolization, prompting the policy of urgent surgery.

Additionally, the most common approach for surgical treatment of infective endocarditis is median sternotomy; however, there is a high possibility of postoperative mediastinitis with a median sternotomy, and some reports recommend a right thoracotomy.^13^ Although reliable verrucous resection and treatment of valvular disease are the highest priorities, a right small thoracotomy should be considered. In our case, we performed MICS and were able to avoid mediastinitis; the patient improved without wound infection.

Vancomycin and ceftriaxone were used for postoperative antibiotic therapy. The antibacterial activity of vancomycin and ceftriaxone against *S. aureus* has been reported to be inferior to that of cefazolin^14^; however, given the multiple cerebral emboli, we chose ceftriaxone due to its good cerebrospinal fluid transfer.^15^ Close observation and skin treatment should be continued; moreover, the patient should be treated for atopic dermatitis in collaboration with a dermatologist.

## 4 CONCLUSION

We encountered a case of a young patient with infective endocarditis in a background of atopic dermatitis. Due to the high risk of embolism, it is necessary to keep in mind that atopic dermatitis can cause infective endocarditis and to make an early diagnosis. It is also important to ensure that appropriate antibiotic therapy and surgery are timed correctly. To prevent recurrence, skin treatment is necessary after surgery, as well as collaboration with a dermatologist.

## AUTHOR CONTRIBUTIONS

**Yamato Tamura:** Conceptualization; data curation; formal analysis; investigation; methodology; project administration; resources; software; supervision; validation; visualization; writing – original draft. **Takehisa Abe:** Supervision; validation; writing – review and editing.

## FUNDING INFORMATION

None.

## CONFLICT OF INTEREST STATEMENT

Yamato Tamura and Takehisa Abe have no conflicts of interest.

## ETHICS STATEMENT

Ethics approval and consent to participate are not applicable for this type of study.

## CONSENT

Written informed consent was obtained from the patient to publish this report in accordance with the journal's patient consent policy.

## Data Availability

The data that support the findings of this study are available from the corresponding author upon reasonable request.
